# Supplementary material for: Look beyond the Mirror: Laparoscopic Cholecystectomy in Situs Inversus Totalis—A Systematic Review and Meta-Analysis (and Report of New Technique)
Source: Diagnostics (Basel). 2022 May 19;12(5):1265. doi: 10.3390/diagnostics12051265 (PMC9140146; doi:10.3390/diagnostics12051265)
Supplement: Supplementary file 1 [file diagnostics-12-01265-s001.zip › diagnostics-1697783-supplementary.pdf]

Table S1: Summary of included cases and reports.

YEAR	AUTHORS	SEX	AGE	DISEASE	DOM. HAND	OP. TECH
1991	CAMPOS, L., SIPES, E.	F	39	AC	R	MIRA
1992	P. GOH, Y. TEKANT, NS SHANG, SS NGOI	M	62	AC		MIRA
1992	SM HUANG, GY CHAU, WY LUI	M	36	CC		MIRA
1992	H. THOMAS TAKEI, J. GARY MAXWELL, THOMAS V. CLANCY, AND ELLIS A. TINSLEY	F	51	CC		MIRA
1993	J. P. MCDERMOTT, P. F. CAUSHAJ	M	66	CHO		MIRA
1996	MALATANI, T.S.	F	25	CC		MIRA
1997	HABIB, Z., SHANAFEY, S., ARVIDSSON, S.	F	45	CC		MIRA
1999	H. DEMETRIADES D. BOTSIOSA C. DERVENIS J. EVAGELOUA S. AGELOPOULOS J. DADOUKIS	F	61	AC	R	MIRA
1999	H. DEMETRIADES D. BOTSIOSA C. DERVENIS J. EVAGELOUA S. AGELOPOULOS J. DADOUKIS	M	37	CC	R	MIRA
2000	KOMBOROZOS, V., PAPOUDOS, M., YANNOPOULOS, P.	M	61	AC	L	MIRA
2001	RAMI J. YAGHAN, FRCS, KAMAL I. GHARAIBEH, FRCS, SAHEL HAMMORI, M.D.2	F	48	CC	R	MIRA
2001	RAMI J. YAGHAN, FRCS, KAMAL I. GHARAIBEH, FRCS, SAHEL HAMMORI, M.D.2	F	38	AC	R	MIRA
2001	M. AL-JUMAILY, FRCS (ED), M. ACHAB, DIS, AND F. HOCHE, CES	F	46	AC		
2001	RAMAMURTHY DONTHI, MD, DAVID J. THOMAS, [...], AND STEVEN P. SCHMIDT, PHD	F	43	CC		MIRA
2001	TARIK ZAFER NURSAL, M.D.,1 ATAÇ BAYKAL, M.D.,2 DÜZGÜN IRET, M.D., 2, AND ÖMER ARAN, M.D.2	F	42	CC	R	MIRA
2002	HONDA, M., TAKESUE, F., YASUDA, M., INUTSUKA, S., NOZOE, T., KORENAGA, D.	M	49	CC	R	MIRA
2002	A. POLYCHRONIDIS, A. KARAYIANNAKIS, S. BOTAITIS, S. PERENTE, AND C. SIMOPOULOS	M	68	CC		MIRA
2003	LM OMS, JM BADIA	F	70	CC	R	MIRF
2003	LM OMS, JM BADIA	M	65	AC	L	MIRF
2003	CAPOV, I., BODO, F., WECHSLER, J., ROVNÁK, J.	F	51	AC	R	MIRA
2003	CAPOV, I., BODO, F., WECHSLER, J., ROVNÁK, J.	F	53	AC	R	MIRA

2004	SUNG-BUM KANG, MD AND HO-SEONG HAN, MD	F	64	AC, CHL		MIRA
2005	SUMIHIRO KAMITANI, YOSIHIRO TSUTAMOTO, KAZUYOSHI HANASAWA, TOHRU TANI	M	76	CC		MIRA
2005	ERKAN, N., AGDENIZ, S., POLAT, A.F., AKSOZ, K., YILMAZ, C., YILDIRIM, M., UNSAL, B.	F	41	CC	R	MIRA
2005	ERKAN, N., AGDENIZ, S., POLAT, A.F., AKSOZ, K., YILMAZ, C., YILDIRIM, M., UNSAL, B.	M	37	AC, CHL	R	MIRA
2005	M. PITIAKOUDIS, A. K. TSAROUCHA, M. KATOTOMICHELAKIS, A. POLYCHRONIDIS & C. SIMOPOULOS	F	47	CC		MIRA
2005	DAMIAN MCKAY* AND GEOFFREY BLAKE	F	32	CC	R	MIRA
2006	UNAL AYDIN, OMER UNALP, PINAR YAZICI, BARIS GURCU, MURAT SOZBILEN, AHMET COKER	M	35	CC	R	
2006	NORMAN ONEIL MACHADO, MS, FRCSED, PRADEEP CHOPRA, MS, FRCSED	F	65	CC	R	MIRA
2006	BORIS KIRSHTEIN, MD,* SOFIE LANTSBERG, MD,W SOLLY MIZRAHI, MD, FACS,* AND LEONID LANTSBERG, MD*	F	51	AC	R	MIRF
2006	AY SHAH, BC PATEL, BA PANCHAL	F	60	CC	R	MIRA
2007	SENTHIL KUMAR, GIUSEPPE FUSAI	F	57	CC	R	MIRA
2007	JAMAL HAMDI, OMAR ABU HAMDAN	M	41	CA	R	MIRA
2008	THEODOROS E. PAVLIDIS, KYRIAKOS PSARRAS, APOSTOLOS TRIANTAFYLLOU, GEORGIOS N. MARAKIS	F	34	AC	R	MIRA
2008	MAURO NEIVA FERNANDES, IVAN NAZARENO CAMPOS NEIVA, FRANCISCO DE ASSIS CAMACHO, LUCAS CROCIATI MEGUINS	F	43	CC	L	MIRA
2009	VELIMEZIS G, ANTONIADES J, FRAGGEDAKIS G, SISAMAKIS G	F	66	CC	R	MIRA
2009	SIMMONS, JON D; MAXWELL, ERICA, MD; VICK, KENNETH D, MD.	F	18	CC	R	MIRA
2009	EISENBERG DAN	M	61	CC	R	MIRA
2010	VIKAS JINDAL, MS, DNB,1 MAHESH C. MISRA, MS, FRCS, FACS,1 VIRINDER K. BANSAL, MS, ASURI KRISHNA, MS,1 RAJESH	F	55	AP, AC	R	MIRA

	PANWAR, MS,1 AND VIMI REWARI, MD2					
2010	VIKAS JINDAL, MS, DNB,1 MAHESH C. MISRA, MS, FRCS, FACS,1 VIRINDER K. BANSAL, MS, ASURI KRISHNA, MS,1 RAJESH PANWAR, MS,1 AND VIMI REWARI, MD2	F	26	AC	R	MIRA
2010	PATLE, N.M., TANTIA, O., SASMAL, P.K., KHANNA, S., SEN, B.	F	36	CC	R	MIRA
2010	PATLE, N.M., TANTIA, O., SASMAL, P.K., KHANNA, S., SEN, B.	F	43	CC	R	MIRA
2010	PATLE, N.M., TANTIA, O., SASMAL, P.K., KHANNA, S., SEN, B.	F	27	CC	R	MIRA
2010	PATLE, N.M., TANTIA, O., SASMAL, P.K., KHANNA, S., SEN, B.	M	48	CC	R	MIRA
2010	PATLE, N.M., TANTIA, O., SASMAL, P.K., KHANNA, S., SEN, B.	F	59	AC	R	MIRA
2010	PATLE, N.M., TANTIA, O., SASMAL, P.K., KHANNA, S., SEN, B.	F	33	CC	R	MIRA
2010	TC HALL, J BARANDIARAN, EP PERRY	M	53	CC	R	MIRA
2011	PAPAZIOGAS, B., KOUTELIDAKIS, I., TSIAOUSIS, P., PARASKEVAS, G., CHATZIMAVROUDIS, G., ATMATZIDIS, S.	F	55	CC	R	MIRA
2011	TOPUZ, O., SOZEN, S., AGACHAN, A.F., TUKENMEZ, M., VURDEM, U.E.	M	50	CC	R	MIRA
2011	EVOLI, LP; MIGLIONICO, L; GRAZIOSI, L; CAVAZZONI, E; BUGIANTELLA, W; DEI SANTI, V; DONINI, A	F	48	AC	L	MIRA
2011	MEHMET ULUDAG, MD, GURKAN YETKIN, MD, ABDULCABBAR KARTAL, MD	M	49	CC	R	SP
2011	MUSTAFA OZSOY, 1 MEHMET FATIH HASKARACA, 1 ALIHAN TERZIOGLU 2	F	65	AC	R	SP
2011	HYUNG JOON HAN1 , SAE BYEOL CHOI1 , CHUNG YUN KIM2 , WAN BAE KIM1 , TAE JIN SONG	M	45	CC	R	SP
2012	HARVINDER SINGH PAHWA, AWANISH KUMAR, ROHIT SRIVASTAVA	F	46	CC	R	MIRA
2012	DEMIRYILMAZ, I., YILMAZ, I., ALBAYRAK, Y., PEKER, K., SAHIN, A., SEKBAN, N.	F	55	CC	R	MIRA
2012	DEMIRYILMAZ, I., YILMAZ, I., ALBAYRAK, Y., PEKER, K., SAHIN, A., SEKBAN, N.	F	51	AC, CHL	R	MIRA
2012	ELBESHRY, T.M., GHNNAM, W.M.	F	24	CC	R	MIRA

2012	LOCHMAN, P., HOFFMANN, P., KOČÍ, J.	F	75	AC	R	MIRA
2012	BOZKURT S, COSKUN H, ATAK T, ET AL.	M	49	CC		SP
2012	MARCUS VINICIUS DANTAS DE CAMPOS MARTINS1,2*, JOSÉ LUIS PANTALEÃO FALCÃO1,2, JAMES SKINOVSKY	F	59	CC		SP
2012	D.R. IUSCO, S. SACCO, I. ISMAIL, S. BONOMI, S. VIRZÌ	F	52	CC	R	MIRF
2013	ARYA, S.V., DAS, A., SINGH, S., KALWANIYA, D.S., SHARMA, A., THUKRAL, B.B.	F	35	CC	R	MIRA
2013	MOHAMMED SULAIMAN ALI, SAAD MUWAFAQ ATTASH	F	43	CC	R	
2013	STOJCEV, Z., DUSZEWSKI, M., BOBOWICZ, M., GALLA, W., MALISZEWSKI, D.	M	47	AC	R	MIRA
2013	SHENG-NING ZHANG*, LI LI, JIANG-HUA RAN, JING LIU, YU LIANG, YANG GAO, AND MIN-JIE YANG	F	50	CC	R	MIRA
2013	ELBERT KHIANGTE1 , IHEULE NEWME2 , KARABI PATOWARY3 , PARTHA PHUKAN4	M	65	CC	R	SP
2014	REDDY, A., PARAMASIVAM, S., ALEXANDER, N., ABHILASH, RAVISANKAR, V., THILLAI, M.	F	45	AC, CHL	R	MIRA
2014	BUTT, M.Q., CHATHA, S.S., GHUMMAN, A.Q., RASHEED, A., FAROOQ, M., AHMED, J.	F	42	CC		MIRA
2014	MOIRANGTHEM, G.S., SINGH, C.A., CHAKRABORTY, G., LOKENDRA, K., PRABHU, T.	F	50	CC	R	MIRA
2014	RAGHUVEER M N, MAHESH SHETTY S, SUNIL KUMAR B B	M	55	CC	R	MIRA
2015	NATTHAWUT PHOTHONG THAWATCHAI AKARAVIPUTH VITOON CHINSWANGWATANAKUL AND ATTHAPHORN TRAKARNSANGA	F	39	CC	R	MIRA
2015	EL-SAADY, AM	F	52	CC	R	MIRA
2015	YOSHIO DEGUCHI, KEITARO MITAMURA, SHUNSUKE OMOTAKA, JUN-ICHI EGUCHI, DAI SAKUMA, MASASHI SATO	M	66	CC	R	SP
2015	SHEIKH SAYIDUL HAQUE , SYED AL FESUNY , MD. BELALUDDIN , S. M. SAKIB KABIR , MEER ISHRAT JAHAN	F	27	CC	R	MIRA
2016	ALSABEK, M.B., ARAFAT, S., ALDIRANI, A.	F	50	CC	L	MIRA

2016	DUNCAN, T.K., WAXMAN, K.	F	42	AC, CHL	AMBI	MIRA
2016	AHMED, Z., KHAN, S.A., CHHABRA, S., YADAV, R., KUMAR, N., VIJ, V., SAXENA, D., TALERA, D.	F	46	AC	R	MIRA
2016	AHMED, Z., KHAN, S.A., CHHABRA, S., YADAV, R., KUMAR, N., VIJ, V., SAXENA, D., TALERA, D.	F	44	CC	R	MIRA
2016	AHMED, Z., KHAN, S.A., CHHABRA, S., YADAV, R., KUMAR, N., VIJ, V., SAXENA, D., TALERA, D.	F	33	CC	R	MIRA
2016	FLEUR BERBERS, SUZANNE VAN LEUVEN, TON K.C.A. VAN ENGELENBURG, RICHARD P.G. TEN BROEK	M	44	AC		MIRA
2016	ROBIN BOTHRA ,AKHILESH SHEKHAWAT	F	45	AC	R	MIRA
2016	ROBERTO MARCELLUS DE BARROS SENA, MARCELO BARROS WEISS, ANA PAULA TEIXEIRA DE ABREU,	F	16	AC		MIRA
2016	SARDAR ARIF	F	42	AC	L	MIRA
2017	NARONGSAK RUNGSAKULKIJ AND PONGSATORN TANGTAWEE	M	32	CC	R	MIRA
2017	JIAN-JUN REN SHU-DONG LI YA-JUN GENG AND RUI XIAO	F	36	CC	R	MIRA
2017	FANSHAWE, AEE; QURASHI, K	F	53	AP	R	MIRF
2017	ANIS HADDAD*, AMINE SEBAI, SOUHAIB ATRI, AMINE DAGHFOUS AND ZOUBEIR BEN SAFTA	M	58	AC	R	MIRF
2017	ANIS HADDAD*, AMINE SEBAI, SOUHAIB ATRI, AMINE DAGHFOUS AND ZOUBEIR BEN SAFTA	M	54	AC	R	MIRF
2017	RAHEEL AHMAD, MUHAMMAD S. SHAFIQUE*, SHEIKH H. AHMAD, SAQIB MEHMOOD, JAHANGIR S. KHAN	F	57	AP		MIRA
2017	AZHAR ALAM, AND ABHIJIT SANTRA	F	20	CC	R	MIRA
2017	DEBORAH MARQUES CENTENO1, TIAGO GALAN DE FRANÇA1, ACÁCIO AUGUSTO CENTENO NETO	F	16	AC	R	MIRF
2017	MEHMET TOLGA KAFADAR, , METIN YALCIN, , YILDIRAY DADUK, , MURAT SOYALP, , MEHMET ALI GOK	F	59	AC	R	MIRA
2017	MEHMET GÖKÇEIMAM, BURAK GÜNEY, EMIN KÖSE, DENIZ TAZEOĞLU, MEHMET CAN AYDIN, KADIR MEKE, SERVET RÜŞTÜ KARAHAN	F	39	AC	R	MIRA
2018	TAKALKAR, Y.P., KORANNE, M.S., VASHIST, K.S., KHEDEKAR, P.G.,	F	50	AC, CHL	AMBI	MIRA

	GARALE, M.N., REGE, S.A., DALVI, A.N.					
2018	RAJINDER SINGH JHOBTA, ARCHIT GUPTA, BALWANT NEGI, KAPIL NEGI	F	23	CC	R	SP
2018	GULZAR AHMAD BHAT* AND DEEPAK GHULLIANI	M	21	CC	R	MIRA
2018	JAE YOOL JANG, M.D., WOOHYUNG LEE, M.D., JINKYU CHO, M.D., CHI-YEONG JEONG, M.D., PH.D., SOON-CHAN HONG, M.D., PH.D.	M	63	CC	R	MIRA
2018	JAE YOOL JANG, M.D., WOOHYUNG LEE, M.D., JINKYU CHO, M.D., CHI-YEONG JEONG, M.D., PH.D., SOON-CHAN HONG, M.D., PH.D.	F	57	AC	R	MIRA
2019	ALKHLAIWY, O., ALMUHSIN, A.M., ZAKARNEH, E., TAHA, M.Y.	M	40	CC	R	MIRA
2019	MOHAMED ALI CHAOUCH, HICHEM JERRAYA, MOHAMED WEJIH DOUGAZ, RAMZI NOUIRA & CHADLI DZIRI	F	48		R	MIRF
2019	MOHAMED ALI CHAOUCH, HICHEM JERRAYA, MOHAMED WEJIH DOUGAZ, RAMZI NOUIRA & CHADLI DZIRI	M	88		R	MIRF
2019	FALAK SHER MALIK, USMAN ISMAT BUTT, WASIM HAYAT KHAN2, SYED MUHAMMAD BILAL2	F	40	CC	R	MIRA
2019	LIANGSHUO HU, MD, YICHAO CHAI, MD, XUE YANG, ZHENG WU, HAO SUN, ZHENG WANG	M	72	AP	R	MIRA
2019	EUN JEONG JANG, M.D., YOUNG HOON ROH, M.D., PH.D	M	37	CC	R	SP
2019	AYAD AHMAD MOHAMMED, SARDAR HASSAN ARIF	M	28	CC	RL	MIRA
2019	BHUPANDER KUMAR CHAWLA, ADITYA CHAWLA	F	30	CC	R	MIRA
2020	MOYON C, M.A., ROJAS, C.L., MOYON C, F.X., AGUAYO, W.G., MOLINA, G.A., OCHOA, C., NEIRA, A.	M	55	AC	R	MIRA
2020	TIANLI DU, ABDELKADER HAWASLI, KAREN SUMME, AHMED A. MEGUID, CHRISTOPHER LAI, MOUTAMN SADOUN	F	56	AC	R	MIRA
2020	BRAÑES, A., PÉREZ, G.	F	79	AC, CHL	R	MIRF
2020	FERNANDO PONCE LEON, MARIANA H. FIORENCIO, CAMILLA P. LEAL, ANDRÉ R. SANTOS	F	61	CC	R	MIRA
2020	PALOMBA, G., DINUZZI, V.P., MANIGRASSO, M., MILONE, M., DE PALMA, G.D. AND APREA, G	F	24	CC	L	MIRA

2021	GELEVSKI, R., JOTA, G., TODOROVIĆ, L., TRAJKOVSKI, G., JOKSIMOVIĆ, V., TRENČIĆ, B.	F	61	CC	R	MIRA
2021	ALIAS, J., ARSHAD, A.W.M., MOHAMAD, I.S., MENG, L.V.	F	58	CC, CHL	R	MIRA
2021	SIMKHADA, S., MALLA, B., SHRESTHA, R.	M	54	CC	R	MIRA
2021	SIMKHADA, S., MALLA, B., SHRESTHA, R.	F	63	CC, CHL	R	MIRF
2021	TIRELLI F, GRIECO M, BIONDI A, BELIA F, PERSIANI R	F	29	CC	R	
2021	HERRERA ORTIZ A, LACOUTURE J C, SANDOVAL MEDINA D, ET AL	F	46	AC	R	MIRA
2021	TAVASSOLI, A., ZANDBAF, T. AND REZAPANAH, A	F	52	CC	R	
2021	TAVASSOLI, A., ZANDBAF, T. AND REZAPANAH, A	F	64	CC	R	
2021	RANJAN R, SAHU KK, PRASAD VS	F	24	CC	R	MIRA
2022	PRESENT CASE REPORT	F	38	CC	R	MIRA
